# A Case of Eclampsia

**Published:** 1862-06

**Authors:** 


### A CASE OF ECLAMPSIA.

May 3d, 1862. Was called in to see Mrs. G., in this city; aged 20; about six months advanced in first pregnancy. Generally healthy and even robust. Never has been sick, except some dysmenorrhœa accompanied with nervous disturbance during the catamenial periods. Married seven months. For some little time past, has been occasionally attacked by slight cramping, with a choking sensation in the throat. No loss of sensation or consciousness, and the disagreeable feelings lasted but for a few minutes. Bowels slightly constipated, but no digestive derangement, save the usual morning sickness.

Ordered a saline laxative, and a mixture of Fl. Ext. Valerianæ, Sp. Ammon. Aromat. and Fl. Ext. Hyoscy.

May 4, 12 M. Sent for in haste. Patient had been in strong and general convulsions since 11 A. M. Unconsciousness; inability to swallow; skin hot and perspiring; face flushed; eyes injected; pulse 110, full and bounding; veins distended upon the temples and arms, as though varicose; respiratory movements irregular and choking; the throat stuffed with mucus; tongue and cheeks swollen, having been lacerated by the teeth during the spasms. There had been no fæcal or urinary evacuations since the day previous, but the bladder was not distended.

A powerful contraction of the uterus would commence about once in six minutes, lasting not more than five or ten seconds, and then giving way entirely, the muscles of the voluntary system would convulse in the most fearful manner.

On examination *per vaginam*, the *os* was found not dilated in the slightest degree, and rigid.

As no medicine could be swallowed, as was proved by repeated efforts, ordered a large enema, for the double purpose of clearing the intestine and acting as emollient to the irritable uterus. At the first convulsion the enema would be thrown out with great force, without any of the other intestinal contents. This was repeated several times without success.

Then sought to put the patient under the influence of Chloroform. It could not be seen that, two ounces, carefully held to the lips and nose, produced the slightest impression. Venesection to perhaps twenty-four ounces. It did not seem to mitigate either the force or frequency of the spasms. The case was so situated that there was no possibility of using the warm bath, the effects of which in such cases I have occasionally seen beneficial.

After the bleeding, however, on recourse to the Chloroform, inhalations, there seemed a little more impressibility, but it could only be said that the paroxysms did not become more violent or frequent. Drew from the bladder about six ounces of high colored and offensive urine.

7. P. M. Dissolved a grain and a half or two grains (not weighed) of Morphine in a couple of ounces of solution of starch, and administered *per rectum*, forcing its retention with a folded napkin. Twenty minutes after the convulsive action began sensibly to diminish. At 8 o'clock, there was no convulsive action, but restlessness at periods of five or ten minutes, which, by abdominal palpation, were found to coincide with uterine contraction. Before 9 o'clock these had pretty much subsided. At 10 o'clock there were symptoms of recurring paroxysms. Repeated the enema. From this time until nine or ten of the next morning, May 5th, she was kept fully under the influence of the Morphine.

I had ordered that at any time during the night, when it was found that she could swallow, she was to take a cathartic composed of ℞ Hydrarg. Sub Chlorid gr. xij, Carb. Sodæ gr. x, Pulv. Ipecac gr. j. M. This was given at about 2 o'clock A. M. Had not operated at 11 A. M. of the 5th.

It was evident that the spasms tended to return with about equal violence as the effects of the Morphine passed off. Ordered a strong enema of Senna and Magnes. Sulph. This passed off at about 1 o'clock, P. M., with abundant evidences of mercurial influence, but with no remarkable amount of fæcal matter. Soon after this evacuation consciousness became more distinctly manifested, and she would make the usual affirmative or negative signs in reply to inquiries. *Os uteri* evidently a very little dilated, but uterine contraction not noticeable.

Ordered diuretic mixture of Acet. Potassæ, Sp. Nit. Dul. and Morph. Sulph.

8 P. M. Has rested quietly. No spasmodic action save occasional choking sensations which soon pass off. Directed mixture continued, and the injection of Morph. and Starch to be repeated in night if necessary—but patient did not require it.

May 6th, 9 A. M. Towards daylight the patient gave evidences of severe pain which the attendants supposed was premonitory of the usual convulsion. But within a very few minutes a little sharp cry astonished both mother and attendants. A six months child barely announced its indignation at its untimely entrance to the world—drew a few idle breaths irregularly and imperfectly, and then gave up its tiny ghost—a couple of hours before my arrival. The placenta had not been thrown off, although the pains were quite severe. It was however readily removed, and several firm clots above it, which seemed to produce much uterine grief.

The after pains continuing quite severe, and wandering suspiciously to other parts of the body, ordered an anodyne and took my leave.

8 P. M. Patient has been tolerably quiet, has taken a little toast water and tea. Troubled with occasional choking sensations, only relieved by sitting up in the bed, a position of course contrary to instructions; occasionally, slight general cramping. Pulse getting a little too hard; skin a little too dry; tongue heavily coated; breath fœtid; and urine scanty, thick and offensive. Repeat mercurial cathartic, and secure its operation in the morning with Liq. Potassæ Citrat.

May 7th, 12 M. Cathartic has operated finely. Lochia free, but not profuse. Milk appearing in the breasts. Has a little appetite, and generally about as well as patients after an ordinary labor.

From this time until the 12th she had no marked symptoms save persistence of the choking sensations, for which I prescribed the usual antispasmodics. It turned out, however, that none were taken. Upon the twelfth, her bowels being constipated; tongue heavily coated; urine scanty, thick and dark; with some febrile excitement and nervous disturbance, ordered a cathartic: ℞ Hydrarg. Sub Chlorid. gr. vj, Pulv. Rhei gr. v, Pulv. Ipecac gr. ss. M. To be taken at bed time. Seidlitz Powder in morning, if necessary.

May 5th. Cathartic had relieved general symptoms, but she still complains of the choking sensation in the throat as troubling her very much. Ordered pills containing each a gr. of Valerianate of Zinc, $\frac{1}{4}$ gr. Ext. Belladonnæ and three gr. Ext. Gentian. One to be taken three times a day. Not forgetting a generous diet and fresh air. The case has presented no more trouble worthy of note.

Bleeding in this case was only of indirect advantage—possibly favoring the action of subsequent agencies. In consequence of displacement of the bandages by the struggles of the patient during my absence, the loss of blood was considerably more than intended. The Chloroform proved inefficient, although the article was a good one and carefully and lavishly exhibited. Morphia finally controlled the convulsive action. The mercurial first showed that the seat of the difficulty was reached, but too late to prevent the premature expulsion of the fœtus. The latter was not to be regretted, for the strong probability is that the mother would have been obliged to run the gauntlet of each recurring monthly period, with all the incident hazard. A.

CHICAGO, May 22d, 1862.

